# Evaluation of Impacts of Cellular Metabolism on the Migration of Ovarian Cancer Cells by Two in Vitro Assays: A Method Comparison Study

**DOI:** 10.31661/gmj.v9i0.1831

**Published:** 2020-08-16

**Authors:** Hossein Ghahremani, Majid Sirati-Sabet, Siamak Salami

**Affiliations:** ^1^Clinical Biochemistry Department, School of Medicine, Shahid Beheshti University of Medical Science, Tehran, Iran

**Keywords:** Ovarian Neoplasms, Neoplastic Stem Cells, Metabolism, Cell Migration, In Vitro Techniques

## Abstract

**Background:**

Alteration of metabolic pathways in cancer cells can intensely modulate their migration as an important step in invasion and metastasis. Ketogenic diet showed some contradictory results in cancer patients. In this study the impact of metabolic reprogramming of A2780CP as a model of ovarian cancer stem-like cells on cell migration by two in vitro methods: wound healing and soft agar colony-forming assays.

**Materials and Methods:**

short term and long term metabolic reprogramming were done by restriction of glucose to 250mg/L with or without enrichment with beta-hydroxybutyrate (5 milimolar) for 48 hours and 30 days, respectively. Wound healing assay was done and the wound ratio was calculated for 24 and 48 hours. Soft agar colony formation assay was also done in treated and control cells. For method comparison, ten biological replicates were analyzed in triplicate.

**Results:**

Migration of A2780CP ovarian cancer stem-like cells were significantly alleviated by long term glucose restriction but no significant changes were observed in short term study. Beta-hydroxybutyrate enrichment did not produce significant impacts on glucose restriction in short or long term studies.

**Conclusion:**

The results of colony formation in soft agar and wound or scratch healing assay were in good correlation and convergence which could be used interchangeably in the investigation of metabolic reprogramming in cancer cells.

## Introduction

Cell migration is a major feature of a malignancy that play chief roles in invasion and metastasis. The migratory potential of cancer cells could be investigated by several methods. Some in vitro methods have been well-established to examine the impacts of different factors or treatments on the migration of cancer cells [1]. Scratch test or wound-healing test is one of the popular migration assay tests which enable the investigator to evaluate the migration of the cancer cells after a manipulation [2-4]. In this test, the speed of healing of the wounded gap shows the migratory potential of the cells. It is a simple and versatile techniques that could be monitored by time to time photography or time-lapse recording. The different tendencies of cancer cells for gap filling by swarming from the wounded edges or by the landing of floating cells make it hard to compare the cells with the different types of migration patterns. Soft agar colony-forming assay is the other test for testing the anchorage-independent growth of cancer cells and eventually reflects the migratory potential of the cancer cells [5,6]. It is a widely used test that has not been affected by the types of migration or growth patterns, but it is a more time-consuming method than wound healing assay. Recent findings revealed that cancer cell migration could also be affected by altered cellular metabolism [7]. Metabolic targeting of cancer cells and metabolic reprogramming showed promising results in some cancers. Beneficial impacts of ketogenic diets (KD) in ovarian cancer have recently reported and a randomized controlled trial revealed that KD may improve physical function, increase energy in patients with ovarian cancer. Many studies suggest ketogenic diets and ketones as a complementary and beneficial treatment to cope with tumor growth and metastasis in a variety of cancers [8-10]. The heterogeneity of metabolic preferences in tumor cells, mainly cancer stem cells(CSCs), and the impact of metabolic reprogramming on the behavior of cancer cells such as migration and metastasis have not been studied yet. In this study, the effect of experimental simulation of nutritional ketosis was investigated on the migration of A2780CP cells as a model of ovarian cancer stem-like cells via wound healing and soft agar colony formation assays.

## Materials and Methods

###  Cell Lines and Chemicals

 The authenticated cisplatin-resistant variant of A2780 human ovarian cancer stem-like cell line, A2780CP (C454) was procured from Iran National Cell Bank(NCBI). A2780CP cells are also cross-resistant to Melphalan, Adriamycin and irradiation. Mycoplasma contamination were ruled out by using a polymerase chain reaction test. Glucose-free DMEM (D5030) and (±)-Sodium 3-hydroxybutyrate (54965) were purchased from Sigma-Aldrich (Merck KGaA, Darmstadt, Germany). Other chemicals and supplements were cell culture grade and obtained from Biosera Europe (Nuaille, France) or other suppliers. No further purification were done. Consumables and cell culture vessels were prochased from a Korean company (SPL Life Sciences Co., Ltd).

###  Metabolic Reprograming 

 For the in vitro stimulation of long term ketogenic diet, cell culture vessels were divided two-three groups, glucose restricted to 250mg/L ( ¼ normal concentration) (G-R), five millimolar beta-hydroxybutyrate enriched glucose restricted to 250mg/L (B-E), and low glucose-DMEM receiving control cells (C). DMEM supplemented with penicillin/streptomycin (100 IU/mL, 100 μg/mL) and FBS (10%) were used, and incubated in a humidified incubator at 37°C and 5% CO2. For short term treatment cells were examined 48 hours after reprogramming and for long term treatment, all subcultures were received the same treatment for one month.

###  Wound Healing

 Wound healing assay was done after short term and long term metabolic reprogramming and a total of 2×105 treated and control cells were spread in monolayer (six-well plate). Then the surfaces of the cultured cells were scratched to form several distinct scratches or wounds. Done on. Subsequently, the cells were supplemented with fresh conditioned medium containing 10% FBS and incubated at cell culture incubator for the next 48 hours. Same wounded area were imaged at different time points (0= just after scratch, 24 and48h at twenty four and forty eight hours, respectively). Later, the wounded areas were computed using microscopic measuring software (Optika Vision Pro, Optika Srl, Ponteranica BG, Italy), and the wound ratio was approximated using the following formula:

$$Wonud\;ratio=\frac{Wounded\;Area\;or\;Endpoint\;Scratched}{Wounded\;Area\;or\;Initial\;Scratched}$$

###  Soft Agar Colony Formation Assay

 Assessment of colony formation was done after short term and long term metabolic reprogramming and. low melting agarose with a concentration of 0.7% containing DMEM was plated in 6-well plates, and the plates were incubated at room temperature for 30 min. Subsequently, 5,000 cells of all treated and control cells were mixed with 0.6% agarose in DMEM medium and were seeded on the top of the solidified base layer. The number of colonies was counted after 3 weeks, using a microscope.

###  Statistical Analyses 

 For method comparison, ten biological repeats were analyzed in triplicate. Data are reported as mean ± SD. The results were statistically analyzed using two-tailed appropriate test (GraphPad Prism, version 8.00) and significant difference was defined as the “p-value” less than 0.05.

## Results

###  Short Term Metabolic Reprogramming

**Figure 1:**
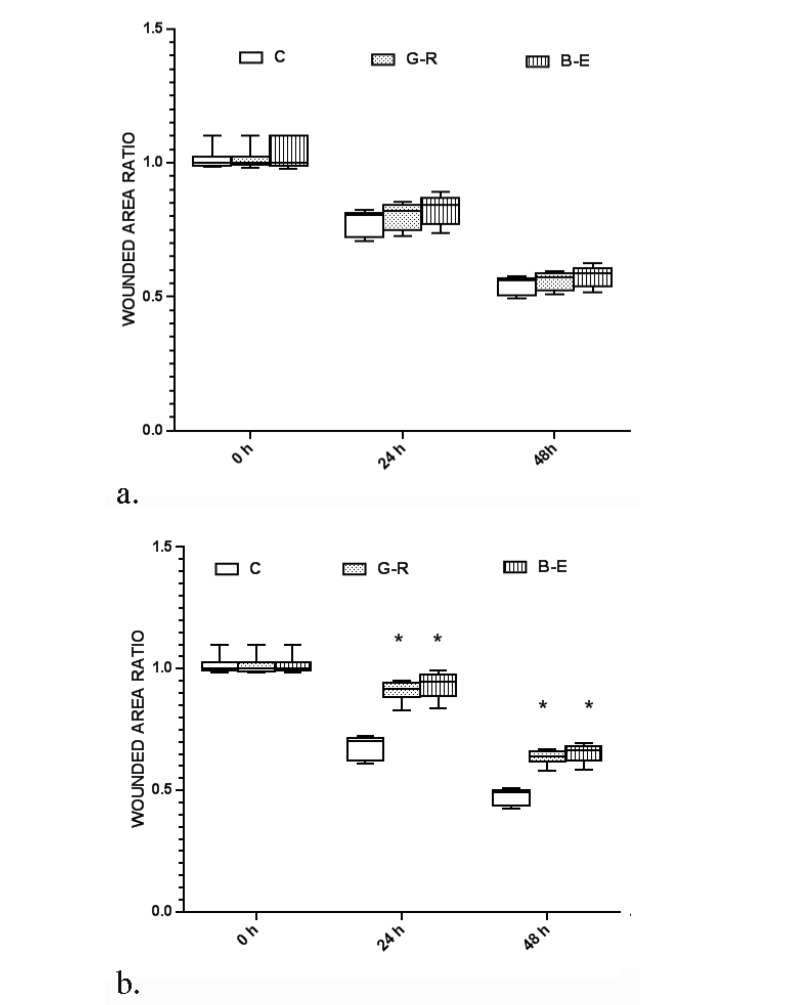
Wound healing assessment of metabolically reprogramed A2780CP cells. a. Short term induction of metabolic changes via restriction of glucose with or without bHB did not show significant changes in 24 or 48 hours. b. Scratch test after a long term metabolic induction by restriction of glucose with or without bHB revealed significant alleviation of A2780CP cells migration in both time-points of 24 and 48 hours. All data represent the mean ± SD of ten biological replicates. *P<0.05

 Short term alteration of available metabolic fuels, by reduction of available glucose with or without supplementation with bHB neither significantly alleviated wound healing at 24 and 48 hours (P=0.7812 and 0.1269, respectively) nor reduced colony formation (P=0.9412 and 0.9734, respectively) (Figure-1a, b). Therefore, short term metabolic alterations of cancer cells have not significantly reflected by stable functional outcomes such as migratory potency or anchorage-independent growth. Such findings are supporting that alteration of migratory potency or anchorage-independent growth is not the early events after changing the available metabolic fuels in ovarian cancer cells.

###  Long Term Metabolic Reprograming

**Figure 2:**
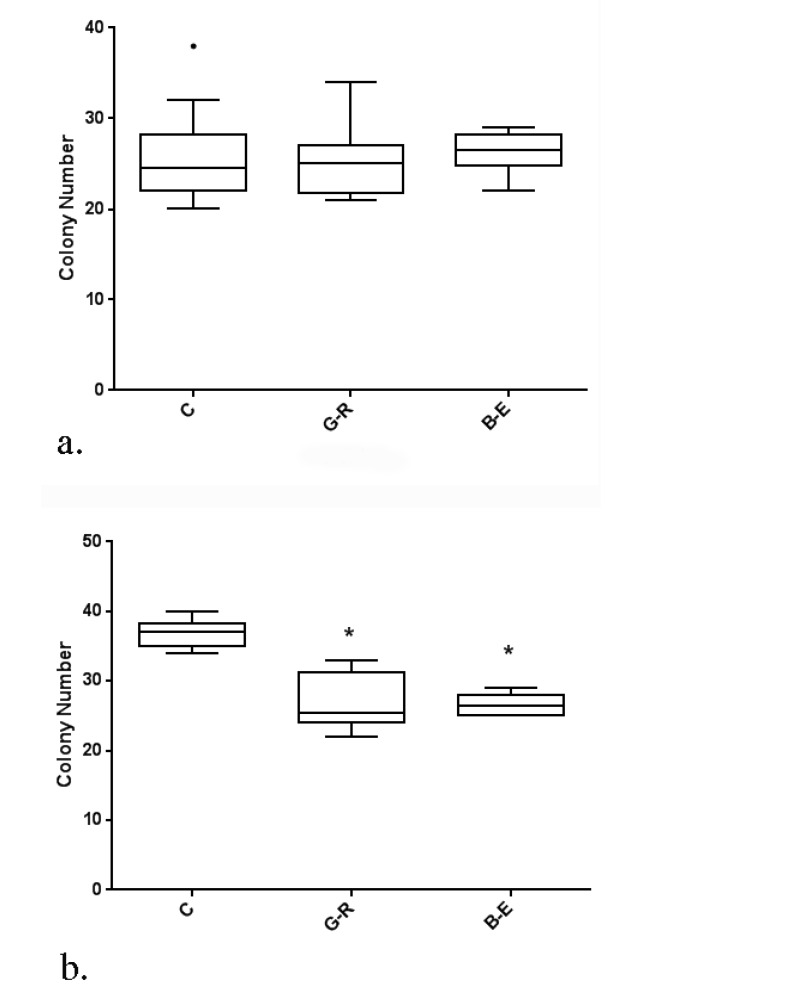
Colony formation in soft agar of metabolically reprogramed A2780CP cells a. Short term restriction of glucose with or without bHB did not induce significant changes in the number of colonies. b. colony formation assay after long term metabolic induction by restriction of glucose with or without bHB showed significant reduction of A2780CP colony numbers as a marker of reduced anchorage independence. All data represent the mean ± SD of ten biological replicates. *P<0.05

 Long term metabolic reprogramming by reduction of glucose availability of the cancer cells to 25% of usual concentration with or without supplementation with 5mM bHB for 30 days revealed that both of the migratory potency or anchorage-independent growth of A2780CP ovarian stem-like cancer cells were significantly impacted. As shown in Figure-2a and b, findings for that glucose restriction with or without bHB significantly reduced migration of A2780CP ovarian stem-like cancer cells in 24 and 48 hours wound healing assay and soft agar colony formation ( P<0.0001 for all). Hence, the stable functional outcome of metabolic reprogramming could be verified by either of soft agar colony formation assay or wound healing assay.

###  Method Comparison

 The results from wound healing tests showed a significant correlation with soft agar colony formation assays in short term (R2=0.683, P=0.035) and long term (R2=0.851, P=0.021) metabolic reprogramming of A2780CP ovarian stem-like cancer cells.

## Discussion

It has been shown that the manipulation of the metabolic machinery of cancer cells dramatically alters the functional outcomes such as migration and invasion [11]. Despite the ability of cancer cells to switch from totally anaerobic to oxidative metabolic pathways in response to certain environmental stressors, glucose is still an important source of cellular ATP for cancer cells. The chief roles of the atypical glycolytic reaction in retaining of the stemness and invasiveness were reported in cancer stem cells (Lin *et al*., 2019). Accordingly, targeting glucose metabolism is a promising approach to eradicating both cancer cells and cancer stem cells. In this regard, several approaches such as intermittent fasting, calorie restriction mimetic drugs and alternative diets such as ketogenic diet have been used in preclinical and clinical studies to prevention and therapy of cancer [12]. In cancer patients, concentration of blood glucose and ketones could be altered to 3.1- 3.8 mM and 2.5-7.0 mM by using a high-fat/low-carbohydrate/adequate-protein ketogenic diet (Seyfried *et al*., 2013). Significant metabolic changes usually occurs within 3 to 4 of ketogenic diet [13]. In this study, we compared the wound healing assay, as a measure of in vitro cell migration assay, and soft agar colony-forming assay, reflecting anchorage-independent cell replication, to examine the impact of short term and long term restriction of glucose with or without bHB supplementation in ovarian cancer stem-like cells, A2780CP. Findings showed that both methods significantly correlate and the short term manipulation did not impact any of those tests. However, a significant correlation was also observed in long term assays indicating that both tests are reliable markers to prove the functional impacts of metabolic reprogramming on in vitro studies and could be used interchangeably. Although the comparison of scratch test and clonogenic assay have not been reported for the impact of short and long term reduction of glucose with or without beta-hydroxybutyrate supplementation in ovarian stem-like cancer cells the similar findings were reported for other kinds of treatments in different cancers [14-16].

## Conclusion

 Wound healing or colony formation in soft agar assays are reliable in vitro test that significantly correlates in confirming the positive impacts or negative responses of metabolic manipulation of the ovarian stem-like cancer cells.

## Acknowledgment

 A part of the article has been extracted from the thesis written by Hossein Ghahremani in the School of Medicine, Shahid Beheshti University of Medical Sciences, Tehran, Iran (registration number: (180). This study was approved by the committee for ethics, School of Medicine, Shahid Beheshti University of Medical Sciences, Tehran, Iran IR.SBMU. MSP.REC.1396.538 and IR.SBMU.SM.REC.1395.571. Part of this study was supported by the research grant (9803-66009803) provided by the deputy of research, Shahid Beheshti University of Medical Sciences.

## Conflict of Interest

 The authors declare that there is no conflict of interest regarding the publication of this article.

**Figure 3:**
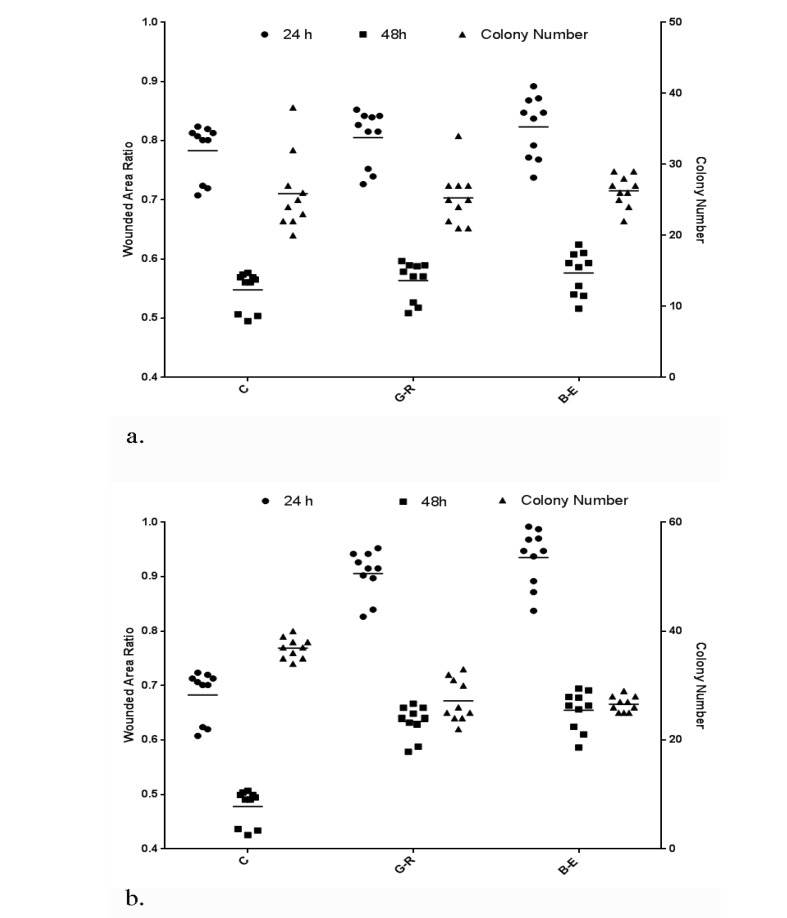
Comparison of colony formation in soft agar with the wound or scratch healing tests for the assessment of metabolic reprogramming in A2780CP ovarian cancer stem-like cancer cells Both methods revealed consistent and convergent results for short (a) and long (b) term metabolic reprogramming with glucose restriction with or without bHB enrichment.
